# Impact of blood glucose levels on the accuracy of urinary N-acety-β-D-glucosaminidase for acute kidney injury detection in critically ill adults: a multicenter, prospective, observational study

**DOI:** 10.1186/s12882-019-1381-3

**Published:** 2019-05-24

**Authors:** Lin Wang, Yujun Deng, Yiling Zhai, Feng Xu, Jinghua Li, Danqing Zhang, Lu Gao, Yating Hou, Xin OuYang, Linhui Hu, Jie Yuan, Heng Ye, Ruibin Chi, Chunbo Chen

**Affiliations:** 1 Department of Intensive Care Unit of Cardiovascular Surgery, Guangdong Cardiovascular Institute, Guangdong Provincial People’s Hospital, Guangdong Academy of Medical Sciences, Guangzhou, 510080 Guangdong Province China; 2 Department of Critical Care Medicine, Guangdong Provincial People’s Hospital, Guangdong Academy of Medical Sciences, 96 Dongchuan Road, Guangzhou, 510080 Guangdong Province China; 3 Department of Emergency Medicine, Shandong University Qilu Hospital, Jinan, 250012 Shandong Province China; 4 Department of Clinical Laboratory, Guangdong Provincial People’s Hospital, Guangdong Academy of Medical Sciences, Guangzhou, 510080 Guangdong Province China; 5 Department of Critical Care Medicine, Guangzhou Nansha Central Hospital, Guangzhou, 511400 Guangdong Province China; 6 Department of Critical Care Medicine, Xiaolan Hospital of Southern Medical University, Zhongshan, 528415 Guangdong Province China

**Keywords:** Acute kidney injury, N-acetyl-β-D-glucosaminidase, Blood glucose levels, Critically ill patients

## Abstract

**Background:**

The performance of urinary N-acetyl-β-D-glucosaminidase (uNAG) for the detection of acute kidney injury (AKI) was controversial. uNAG is positively correlated with blood glucose levels. Hyperglycemia is common in the critically ill adults. The influence of blood glucose levels on the accuracy of uNAG in AKI detection has not yet been reported. The present study evaluated the effect of blood glucose levels on the diagnostic accuracy of uNAG to detect AKI.

**Methods:**

A total of 1585 critically ill adults in intensive care units at three university hospitals were recruited in this prospective observational study. uNAG, serum glucose, and glycosylated hemoglobin (HbA1c) were measured at ICU admission. Patients were categorized based on the history of diabetes and blood glucose levels. The performance of uNAG to detect AKI in different groups was assessed by the area under the receiver operator characteristic curve.

**Results:**

Four hundred and twelve patients developed AKI, of which 109 patients were severe AKI. uNAG was significantly correlated with the levels of serum glucose (*P* < 0.001) and HbA1c (*P* < 0.001). After stratification based on the serum glucose levels, no significant difference was observed in the AUC of uNAG in detecting AKI between any two groups (*P* > 0.05). Stratification for stress hyperglycemic demonstrated similar results.However, among non-diabetic patients, the optimal cut-off value of uNAG for detecting AKI was higher in stress hyperglycemic patients as compared to those without stress hyperglycemia.

**Conclusions:**

The blood glucose levels did not significantly affect the performance of uNAG for AKI detection in critically ill adults. However, the optimal cut-off value of uNAG to detect AKIwas affected by stress hyperglycemia in non-diabetic patients.

**Electronic supplementary material:**

The online version of this article (10.1186/s12882-019-1381-3) contains supplementary material, which is available to authorized users.

## Key messages

- Admission serum glucose and HbA1c levels were positively correlated with uNAG.
- Admission serum glucose and HbA1c levels did not have a significant impact on the discrimination accuracy of uNAG for AKI.
- The optimal cut-off value of uNAG to detect AKI could be affected by stress hyperglycemia in non-diabetic patients.

## Background

Acute kidney injury (AKI) is a frequent occurrence [1–3] and associated with morbidity and mortality [4–6], especially in intensive care unit (ICU) [7, 8]. Early intervention may avoid the heavy burden associated with AKI due to its long-term effects [9]. Therefore, the timely identification of AKI is an urgent requisite.

Urinary N-acetyl-β-D-glucosaminidase (uNAG), originating from the lysosomes of the proximal tubule cells of the kidney, is a sensitive marker for AKI in clinical practice. Since the large size of the uNAG molecule impedes its renal filtration, and high levels of uNAG are unlikely to originate from a non-renal source [10, 11]. Recent studies indicated that uNAG is valuable in the early diagnosis of AKI [1, 12–14]. However, some studies reported a satisfactory discrimination of uNAG in the early detection of AKI in various patient populations [1, 13], while other studies found that uNAG had poor-to- moderate discrimination ability for AKI [15, 16]. These contradictory findings limit the application of uNAG in the early detection of AKI in clinical practice.

Notably, previous studies found that the concentrations of uNAG were positively correlated with blood glucose levels [17–20]. A previous study [20] demonstrated that the level of uNAG increases rapidly due to blood glucose fluctuation. It is possible that increased glucose reabsorption of renal tubule excited the polyol pathway activity, which cause sorbitol accumulation and osmotically tissue damage [21]. Consequently, the damage of renal tubule increased the excretion of uNAG. ICU patients were usually associated with various clinical comorbidities, especially impaired glucose metabolism. Moreover, hyperglycemia, which is common in critically ill patients [22], has been reported to be associated with AKI [23]. However, the effect of blood glucose levels on the performance and threshold of uNAG in detecting AKI has not yet been elucidated.

Therefore, we undertook a multicenter, prospective, observational study to assess the performance of uNAG for AKI detection in critically ill adults classified according to the history of diabetes and blood glucose levels in real- world clinical practice.

## Methods

### Protocol design and study population

Three general ICUs was included in this prospective observational study. Patients admitted to our ICUs between October 2014 and July 2016 were enrolled. Exclusion criteria were known the refusal of consent, age < 18 years, pregnancy, unavailability of the urine sample, hemodialysis or peritoneal dialysis prior to enrollment or end-stage renal disease (ESRD), nephrectomy or renal transplantation. The protocol was followed according to that of the Strengthening the Reporting of Observational Studies in Epidemiology [24] and Standards for Reporting Diagnostic Accuracy [25] criteria. The current study received the approval of the local institutional review board. Written informed consent was obtained from each participant or a family member at the time of enrollment.

### Specimen and data collection

At the time of ICU admission, urine and blood samples were collected immediately. For patients from the participating hospital, urine samples were shipped by cold chain transportation and examined at the clinical laboratory of the Guangdong Provincial People’s Hospital within 24 h after collection. We measured the level of serum creatinine (sCr), uNAG, serum glucose, and glycosylated hemoglobin (Hb1Ac) at the time of ICU admission. uNAG and HbA1c were measured upon ICU admission. sCr and serum glucose were measured at ICU admission and daily as a part of routine clinical care during ICU stay.

We prospectively collected the demographic and clinical characteristics of each patient, including sex, age, body mass index (BMI), preexisting chronic conditions, sepsis, the categories of diseases, Acute Physiology and Chronic Health Evaluation II score (APACHE II), previous application of antidiabetic drugs, baseline serum creatine, baseline estimated glomerular filtration rate (eGFR), and the outcomes. The eGFR of patient was calculated using the abbreviated Modification of Diet in the Renal Disease formula [26]. The hourly urine output was also recorded.

### Biomarker assays

Creatinine, uNAG, and serum glucose were analyzed using the UniCel DxC 800 Synchron System (Beckman Coulter, Brea, CA, USA). The values of uNAG were normalized to that of the urinary creatinine concentrations. The coefficients of interassay and intraassay variation for uNAG were both ≤10%. HbA1c was measured using the D-10™ Hemoglobin Analyzer (Bio-Rad, Hercules, CA, USA). The normal range for HbA1c was 3.8–18.5%.

### Data definitions

AKI was determined based on the criteria of Kidney Disease Improving Global Outcomes Clinical Practice Guidelines [27]: sCr levels increased exceed 0.3 mg/dL (26.5 μmol/L) from baseline within 48 h, or more than 1.5-fold increase in sCr levels from baseline within 7 days, or urine output < 0.5 mL/kg/h for 6 h. KDIGO stage 2 or stage 3 within 7 days after ICU admission were considered as severe AKI. The baseline sCr was defined as following rules in sequence as described in previous study [28]: if patients had serum creatine value before ICU admission, (1) the most recent pre-ICU value (between 30 and 365 days before ICU admission); (2) for patients aged < 40 years, a stable pre-ICU value > 365 days before ICU admission (stable defined as within 15% of the lowest ICU measurement); (3) pre-ICU value (> 365 days before ICU admission) and less than the initial sCr at ICU admission; (4) a pre-ICU value (between 3 and 39 days before ICU admission) ≤ initial sCr at the time of admission to ICU and not distinctly in AKI; if patients did not have serum creatine value before ICU admission, (5) the lowest sCr upon initial admission value, the final ICU value, or the minimum value at follow-up unto 365 days.

### Stratification protocol

All of the groups were preset. To evaluate the performance of uNAG for AKI detection with respect to the admission serum glucose in various degrees, the patients were stratified into five groups as described previously [29]: < 110 mg/dL, 110 to < 140 mg/dL, 140 to < 170 mg/dL, 170 to < 200 mg/dL, and ≥ 200 mg/dL. Patients were also divided into five quintiles based on the levels of serum glucose at the time of admission.

In order to detect the impact of HbA1c levels and history of diabetes on the accuracy of uNAG, patients were additionally stratified into four groups as described previously [30]: recognized diabetes (previous diagnosis of diabetes), undetected diabetes (with HbA1c ≥6.5% but no previous diagnosis of diabetes), prediabetes (5.7% ≤ HbA1c < 6.5% but no previous diagnosis of diabetes), and normal glycemic status (HbA1c < 5.7% but no previous diagnosis of diabetes). Patients were classified as having diabetes if they were diagnosed with diabetes or at least 1 prescription for insulin or an oral antidiabetic agent. Patients without a known history of diabetes and an HbA1c < 6.5% were defined as non-diabetic patients. The recognized and undetected diabetes were categorized as diabetic patients. Stress hyperglycemia was defined as serum glucose levels exceeding the threshold of 200 mg/dL in non-diabetic patients, in accordance with the consensus statement [31]. Likewise, diabetic patients were divided into two subgroups according to abovementioned threshold of serum glucose. The diagnosis of sepsis was defined according to the American College of Chest Physicians/Society of Critical Care Medicine Consensus Conference Committee guidelines [32].

### Statistical analysis

We calculated the sample size based on AKI incidence of 26.86%, which we determined in a chart review of 350 critical patients (unpublished). According to the previous studies [33, 34], we estimated that a sample size requirement of 1415 patients was required, with a two-sided test (α error = 5%; power = 80%). With an estimated loss of 10%, the final sample required will be 1557 patients at least. The Kolmogorov–Smirnov test was used to determine the normal distribution of each variable. Continuous variables were presented as means ± standard deviation and medians (interquartile range). The categorical variables were expressed as counts and proportions. The non-normally distributed continuous variables were compared by Wilcoxon rank-sum test or Kruskal–Wallis test for one-way analysis of variance. To compare the categorical variables, the chi-square test or Fisher’s exact test was used. Bivariate correlation analysis was performed to examine the correlation between two variables. Multivariate linear regression was used to search for factors independently associated with uNAG. The performance of uNAG was determined using the receiver operating characteristic (ROC) curve analysis. The differences between AUC were tested by Hanley–McNeil method [35]. The optimal cut-off values for AKI detection were defined for individual biomarkers using Youden’s index based on which the sensitivity and specificity were calculated.

All statistical tests were 2-tailed, and *P* < 0.05 was considered statistically significant. All analyses were performed using SPSS 13.0 (SPSS, Chicago, IL, USA) or MedCalc version 12.5.0 (MedCalc Software, Ostend, Belgium) software.

## Results

### Patient characteristics

**Fig. 1:**
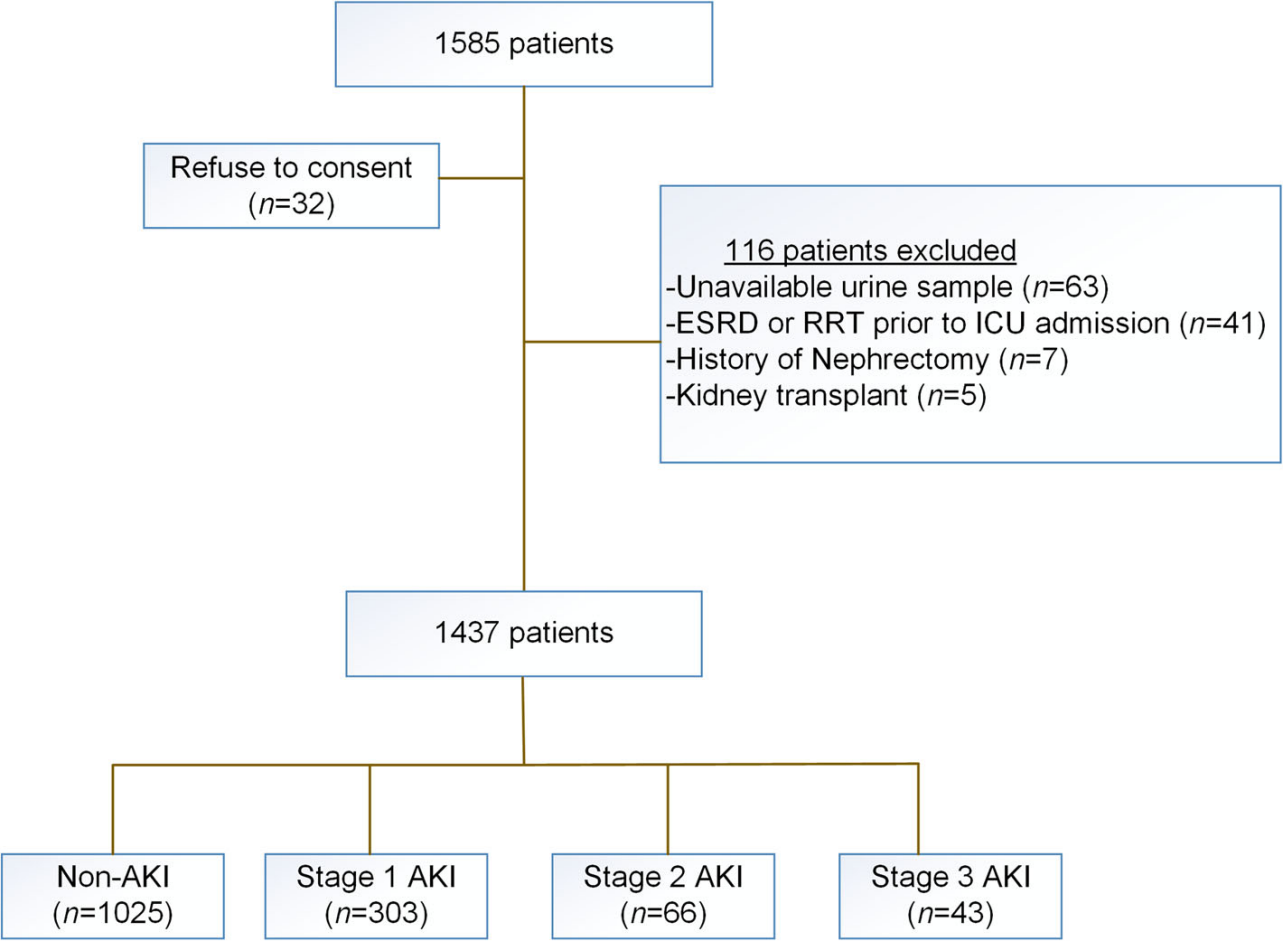
Flow-chart of the critically ill patients. ESRD, End-stage renal disease; RRT, Renal replacement therapy; ICU, Intensive care unit; AKI, Acute kidney injury

Of 1585 consecutive admitted patients from three general ICUs, 148 patients (9.3%) were excluded according to the exclusion criteria (Fig. 1). Of the remaining 1437 subjects, AKI was diagnosed in 412 patients (28.7%). Table 1 presents the baseline clinical data and outcomes of the enrolled patients. Compared to the non-AKI patients, those with AKI were older with high rates of preexisting clinical conditions, including diabetes mellitus (DM), hypertension, and chronic kidney disease (CKD). Diabetes was presented in 225 patients of the entire cohort, including 127 undetected diabetes patients. Patients with AKI had higher concentrations of sCr and serum glucose at the time of ICU admission. Moreover, the concentrations of uNAG were significantly higher in the AKI group than in the non-AKI group.

**Table 1:** Baseline clinical data and outcomes

Characteristics	Non-AKI (*n* = 1025)	AKI (*n* = 412)	*P* value
Demographic variables			
Age, years	52.0 (41.0–62.0)	59.0 (45.0–71.0)	< 0.001
Male sex, *n* (%)	521 (50.8)	247 (60.0)	0.032
BMI, kg/m^2^	22.2 (21.5–23.1)	22.4 (21.5–23.4)	0.223
Preexisting clinical conditions			
Hypertension, *n* (%)	169 (16.5)	137 (33.3)	< 0.001
DM, *n* (%)	117 (11.4)	108 (26.2)	< 0.001
CKD, *n* (%)	16 (1.6)	46 (11.2)	< 0.001
Sepsis, *n* (%)	157 (15.3)	204 (49.5)	< 0.001
Previous antidiabetic drugs, *n* (%)			
α-glucosidase inhibitors, *n* (%)	12 (1.2)	6 (1.5)	0.662
Insulin secretagogues, *n* (%)	10 (1.0)	11 (2.7)	0.016
Thiazolidinediones, *n* (%)	1 (0.1)	3 (0.7)	0.041
Metformin, *n* (%)	15 (1.5)	14 (3.4)	0.431
Insulin, *n* (%)	7 (0.7)	12 (2.9)	0.235
Admission type, *n* (%)			< 0.001
Elective surgical, *n* (%)	798 (77.9)	194 (47.1)	
Emergency surgical, *n* (%)	94 (9.2)	67 (16.3)	
Medical, *n* (%)	133 (13.0)	151 (36.7)	
Baseline serum creatinine, mg/dl	0.69 (0.59–0.83)	0.71 (0.56–0.93)	0.145
Baseline eGFR, ml/minute/1.73 m^2^	110.62 (95.27–132.95)	109.50 (83.36–139.68)	0.146
Serum creatinine at admission, mg/dl	0.77 (0.64–0.92)	1.04 (0.80–1.33)	< 0.001
uNAG at admission, U/g Cr	22.55 (13.26–37.75)	35.46 (21.26–60.96)	< 0.001
Serum glucose at admission, mg/dl	120.24 (102.74–146.25)	143.19 (117.90–180.45)	< 0.001
HbA1c at admission, %	5.60 (5.30–6.00)	5.80 (5.40–6.30)	< 0.001
APACHE II score	10 (8–14)	16 (10–23)	< 0.001
UP, ml/kg/h	2.05 (1.57–2.69)	1.86 (1.24–2.62)	< 0.001
Outcomes			
Length of ICU stay, days	2 (2–4)	4 (2–9)	< 0.001
Length of hospital stay, days	10 (8–15)	13 (8–21)	< 0.001
RRT during ICU stay, *n* (%)	3 (0.3)	21 (5.1)	< 0.001
ICU mortality, *n* (%)	28 (2.7)	51 (12.4)	< 0.001
In-hospital mortality, *n* (%)	37 (3.6)	57 (13.8)	< 0.001

*AKI* acute kidney injury, *BMI* body mass index, *DM* diabetes mellitus, *CKD* chronic kidney disease, defined as baseline estimated glomerular filtration rate < 60 ml/min/1.73 m^2^; eGFR, estimated glomerular filtration rate, *uNAG* urinary N-acetyl-glucosaminidase, the values of uNAG were normalized to urinary creatinine concentration, *Cr* creatinine concentration, *HbA1c* glycosylated hemoglobin, *APACHE II* Acute Physiology and Chronic Health Evaluation score, *UP* urine production first 24 h after admission, *ICU* intensive care unit, *RRT* renal replacement therapy. *P* value for global comparisons among groups by Kruskal-Wallis and chi-square tests for continuous and categorical variables, respectively

### Baseline factors related to uNAG

Bivariate correlation analysis (Table 2) showed that a high level of uNAG was associated with older age, higher APACHE II score, and a remarkable change between the levels of initial sCr and baseline sCr. In addition, a positive correlation was established between uNAG and diabetes mellitus (*r* = 0.124, *P* < 0.001) as well as HbA1c (*r* = 0.147, *P* < 0.001) and a positive association between uNAG and admission glucose (*r* = 0.196, *P* < 0.001). In multivariate regression (Table 3), age (standardized β = 0.187, *P* < 0.001), baseline sCr (standardized β = − 0.093, *P* < 0.001), △sCr (standardized β = 0.089, *P* < 0.001), and admission glucose (standardized β = 0.147, *P* < 0.001) were significantly correlated with the levels of uNAG.

**Table 2:** Factors associated with uNAG by bivariate correlation analysis

Variables	uNAG	
	*R*	*P*
Scr at admission (mg/dl)	−0.079	0.003
Baseline Scr (mg/dl)	−0.158	< 0.001
ΔScr (mg/dl)	0.087	0.001
Age (years)	0.295	< 0.001
Diabetes mellitus	0.124	< 0.001
Serum glucose at admission (mg/dl)	0.196	< 0.001
HbA1c at admission (%)	0.147	< 0.001
APACHE II score	0.055	0.036

*uNAG* urinary N-acetyl-glucosaminidase, *Scr* serum creatinine concentration, *ΔScr* the change between admission Scr and baseline Scr, *HbA1c* glycosylated hemoglobin, *APACHE II* Acute Physiology and Chronic Health Evaluation score

**Table 3:** Factors associated with uNAG in multivariate linear regression

Independent Variables^a^	uNAG	
	Standardized β	*P*
Baseline Scr (mg/dl)	−0.093	< 0.001
ΔScr (mg/dl)	0.089	0.001
Age (years)	0.187	< 0.001
Serum glucose at admission (mg/dl)	0.147	< 0.001

^a^Independent variables included age, baseline Scr, admission Scr, ΔScr, diabetes mellitus, admission serum glucose, HbA1c. Variables not listed in the table were removed from the stepwise analysis. Adjusted R square 0.08. Baseline Scr, baseline serum creatinine; ΔScr, the change between admission Scr and baseline Scr

### Performance of uNAG for AKI detection with respect to serum glucose stratification

**Fig. 2:**
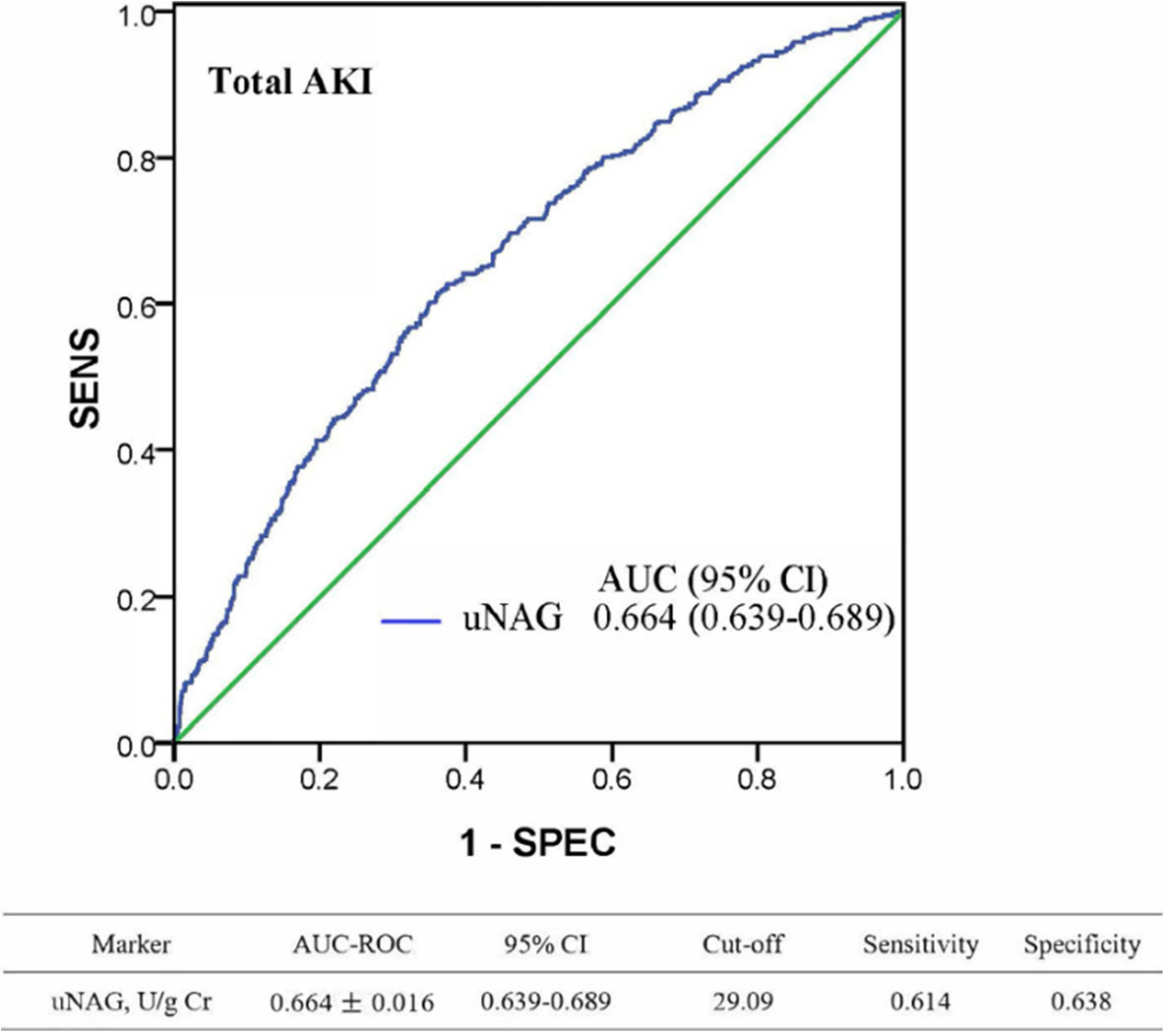
ROC analysis for the ability of uNAG to detect total AKI. AKI, acute kidney injury; uNAG, urinary N-acetyl-glucosaminidase; AUC, area under the receiver operating characteristic curve; 95% CI, 95% confidence interval

ROC analysis revealed that uNAG detected total AKI with poor-to-moderate discrimination capability (Fig. 2). The AUC-ROC value of uNAG for detecting total AKI was 0.664 [95% confidence interval: 0.639–0.689]. To evaluate the impact of admission serum glucose levels on the accuracy of uNAG to detect AKI, an AUC-ROC value was calculated in each group of admission serum glucose: < 110 mg/dL, 110 to < 140 mg/dL, 140 to < 170 mg/dL, 170 to < 200 mg/dL, and > 200 mg/dL (Table 4); no significant difference was observed in AUCs between any two groups. Similarly, no significant difference was observed in AUCs for severe AKI detection between any two groups (*P* > 0.05). In addition, we evaluated the AUCs of uNAG on the diagnosis of total AKI and severe AKI in each quintile of the level of admission serum glucose (Additional file 1: Table S1). There was no significant difference in the AUC between any two sub- cohorts.

**Table 4:** AUCs for AKI stratified according to admission serum glucose concentrations

Glucose (mg/dl)	AKI *(n, %)*	AUC-ROC	95% CI	Cut-off (U/g Cr)	Sensitivity	Specificity
Total AKI (*n* = 412)						
<110 (*n* = 448)	77 (17.2)	0.643 ± 0.034	0.597–0.687	21.04	0.727	0.496
110 to < 140 (*n* = 468)	119 (25.4)	0.645 ± 0.029	0.600–0.689	28.47	0.546	0.673
140 to < 170 (*n* = 275)	93 (33.8)	0.639 ± 0.036	0.579–0.696	32.89	0.613	0.654
170 to < 200 (*n* = 123)	51 (41.5)	0.634 ± 0.052	0.543–0.719	29.80	0.726	0.569
≥ 200 (*n* = 123)	72 (58.5)	0.651 ± 0.052	0.560–0.735	28.03	0.778	0.549
Severe AKI (*n* = 109)						
<110 (*n* = 448)	18 (4.0)	0.703 ± 0.068	0.658–0.745	38.37	0.667	0.770
110 to < 140 (*n* = 468)	27 (5.8)	0.756 ± 0.048	0.714–0.794	31.65	0.778	0.687
140 to < 170 (*n* = 275)	28 (10.2)	0.679 ± 0.049	0.621–0.734	33.95	0.750	0.615
170 to < 200 (*n* = 123)	13 (10.6)	0.680 ± 0.086	0.590–0.762	49.25	0.616	0.736
≥200 (*n* = 123)	23 (18.7)	0.703 ± 0.054	0.614–0.782	35.01	0.870	0.560

*AUC* area under the receiver operating characteristic curve, *AKI* acute kidney injury, *n* sample size, 95% CI, 95% confidence interval

Total AKI:

< 110 mg/dl versus 110 to < 140 mg/dl Z = 0.045, *P* = 0.964

< 110 mg/dl versus 140 to < 170 mg/dl Z = 0.081, *P* = 0.935

< 110 mg/dl versus 170 to < 200 mg/dl Z = 0.145, *P* = 0.885

< 110 mg/dl versus ≥200 mg/dl Z = 0.129, *P* = 0.897

110 to < 140 mg/dl versus 140 to < 170 mg/dl Z = 0.131, *P* = 0.895

110 to < 140 mg/dl versus 170 to < 200 mg/dl Z = 0.185, *P* = 0.853

110 to < 140 mg/dl versus ≥200 mg/dl Z = 0.102, *P* = 0.919

140 to < 170 mg/dl versus 170 to < 200 mg/dl Z = 0.079, *P* = 0.937

140 to < 170 mg/dl versus ≥200 mg/dl Z = 0.192, *P* = 0.848

170 to < 200 mg/dl versus ≥200 mg/dl Z = 0.232, *P* = 0.816

Severe AKI:

< 110 mg/dl versus 110 to < 140 mg/dl Z = 0.635, *P* = 0.525

< 110 mg/dl versus 140 to < 170 mg/dl Z = 0.287, *P* = 0.774

< 110 mg/dl versus 170 to < 200 mg/dl Z = 0.210, *P* = 0.834

< 110 mg/dl versus ≥200 mg/dl Z = 0, *P* = 1

110 to < 140 mg/dl versus 140 to < 170 mg/dl Z = 1.125, *P* = 0.261

110 to < 140 mg/dl versus 170 to < 200 mg/dl Z = 0.773, *P* = 0.440

110 to < 140 mg/dl versus ≥200 mg/dl Z = 0.734, *P* = 0.463

140 to < 170 mg/dl versus 170 to < 200 mg/dl Z = 0.010, *P* = 0.992

140 to < 170 mg/dl versus ≥200 mg/dl Z = 0.331, *P* = 0.740

170 to < 200 mg/dl versus ≥200 mg/dl Z = 0.227, *P* = 0.820

### Performance of uNAG for AKI detection in stress hyperglycemia stratification

Among the non-diabetic patients, AUCs was calculated as 0.679 and 0.645 in patients with and without stress hyperglycemia, respectively (Table 5). The optimal cut-off value of uNAG for total AKI detection between stress hyperglycemia and non-hyperglycemia patients was markedly different (43.86 and 29.80 U/g Cr, respectively). In addition, the median concentration of uNAG for AKI patients with stress hyperglycemia was 46.15 U/g Cr (29.89–63.13), which was significantly different from non-stress hyperglycemia patients with the value 32.98 U/g Cr (19.10–57.08) (Additional file 2: Figure S1). Furthermore, uNAG exhibited marked difference optimal cut-off value for detecting severe AKI between these two subgroups (44.74 and 31.65 U/g Cr, respectively). However, the optimal cut-off values of uNAG for detecting AKI in diabetic patients between these abovementioned subgroups were similar (Additional file 3: Table S2).

**Table 5:** AUCs for AKI stratified according to admission serum glucose in non-diabetic patients

Glucose (mg/dL)	AKI (*n*, %)	AUC-ROC	95% CI	Cut-off (U/g Cr)	Sensitivity	Specificity
Total AKI (*n* = 304)						
< 200 (*n* = 1158)	276 (23.8)	0.645 ± 0.019	0.617–0.673	29.80	0.554	0.661
≥ 200 (*n* = 54)	28 (51.9)	0.679 ± 0.074	0.538–0.799	43.86	0.607	0.769
Severe AKI (*n* = 74)						
< 200 (*n* = 1158)	65 (5.6)	0.715 ± 0.033	0.688–0.740	31.65	0.708	0.657
≥ 200 (*n* = 54)	9 (16.7)	0.698 ± 0.085	0.557–0.815	44.74	0.778	0.689

*AUC* area under the receiver operating characteristic curve, *AKI* acute kidney injury, *n* sample size, 95% CI*,* 95% confidence interval

Total AKI:

≥200 mg/dL versus < 200 mg/dL Z = 0.444, *P* = 0.657

Severe AKI:

≥200 mg/dL versus < 200 mg/dL Z = 0.186, *P* = 0.852

### Performance of uNAG for AKI detection in HbA1c stratification

To evaluate the influence of HbA1c levels and history of diabetes on the performance of uNAG in the detection of AKI; the level of HbA1c at the time of ICU admission was measured. Patients were divided into four groups according to the HbA1c levels and history of diabetes (Additional file 4: Table S3). Patients with a known previous history of diabetes were defined as recognized diabetes. Patients without known previous history of diabetes were further divided into three groups. The AUC for total AKI detection was calculated as 0.675 in a sub-cohort of recognized diabetes. In patients without a known previous history of diabetes, the AUC was calculated as 0.649 in the sub-cohort with HbA1c ≥6.5%, 0.645 in the sub-cohort with 5.7% ≤ HbA1c < 6.5%, and 0.659 in the sub-cohort with HbA1c < 5.7%, respectively. The AUC for uNAG in detecting severe AKI in each group were as follows: 0.731 in group of recognized diabetes, 0.704 in group of unrecognized diabetes, 0.700 in group with 5.7% ≤ HbA1c < 6.5% and 0.734 in group with HbA1c < 5.7%. In addition, we also evaluated the AUCs of uNAG on the discrimination ability of AKI in each quartile of HbA1c levels (Additional file 5: Table S4). Similar results were observed, and no significant difference was observed in the AUC for AKI or severe AKI detection between any two groups.

## Discussion

In this large-scale multicenter prospective real-world study, we did not find any significant impact of blood glucose levels regarding the performance of uNAG for AKI detection in critically ill adults. However, the optimal cut-off value of uNAG to detect AKI was affected by stress hyperglycemia in non-diabetic patients.

uNAG is an early tubular damage biomarker that can predict AKI and adverse outcomes [36, 37]. Although an increased uNAG excretion reflects the acute tubular injury [38], uNAG levels are also elevated in different conditions other than AKI, such as diabetes mellitus [39]. uNAG was detected as increased in the early stages of diabetes mellitus even before any clinical evidence of renal involvement was presented [40]. Recent studies revealed that blood glucose levels might increase the excretion of uNAG [17]. On the other hand, uNAG might reflect the glycemic control in insulin-dependent diabetic patients [41, 42]. Another study found a significant correlation between high uNAG values and HbA1c [43]. Moreover, diabetes and hyperglycemia, both common in critically ill patients [22, 23], are associated with the development of AKI [29, 44, 45]. Therefore, we undertook this prospective study to evaluate the effect of blood glucose levels on the performance of uNAG to identify AKI in ICU patients.

Herein, we found significant but weak correlations between admission serum glucose level and uNAG and between HbA1c and uNAG. However, the present study showed that uNAG did not yield any significant difference in AUC for AKI detection among different blood glucose levels, either stratified based on the admission serum glucose or HbA1c. These results strongly suggested that blood glucose did not interfere with the performance of uNAG in the detection of AKI in critically ill adults. Thus, our findings supported the clinical applicability of uNAG in patients with diabetes or prediabetes. To the best of our knowledge, the present study illustrated for the first time that there was no significant impact of blood glucose on the diagnostic accuracy of uNAG in the detection of AKI in the ICU settings based on a large-scale cohort.

The present study did not display a significant discrimination ability of uNAG among patients with different glucose levels. However, the optimal cut-off value of uNAG could be affected by stress hyperglycemia among non-diabetic patients. The current study indicated that the optimal cut-off value of uNAG to detect AKI was markedly higher in those with stress hyperglycemia as compared to without stress hyperglycemia. Thus, this characteristic should be considered when using uNAG to identify AKI in stress hyperglycemia patients. Previous studies [17, 20] reported a rapid change in the uNAG levels in response to the short-term fluctuation of blood glucose, which might be attributed to stress hyperglycemia-induced stimulation of uNAG production in non-diabetic patients. However, a similar result was not observed in the diabetic cohort. The possible reason may be that, in diabetic patients, the elevated blood glucose concentrations is partly due to poor glycemic control but not reflecting a short-term increment of blood glucose. This result implies that the most reasonable cut-off point for stress hyperglycemia in diabetic cohort needs to be established.

Nevertheless, the present study has some limitations. Firstly, we used uNAG to detect the total AKI rather than predict AKI, ascribing to the relatively small number of new AKI cases. Secondly, we used the admission serum glucose level rather than continuous blood glucose variability to evaluate the hypothesis. Thus, additional studies are essential for substantiating the results.

The advantages of this study include its appropriate sample size and a multicenter prospective design that allow the retrieval of real-world data and potentially better data quality as compared to the retrospective designs. Due to the stability of uNAG [46], specific guidelines for urine sample collection, transportation, and unified measurement strategy were followed for uNAG, which is expected to have reduced the systemic errors.

## Conclusion

In this multicenter, prospective, observational study, we found that blood glucose levels might not exert a significant impact on the performance of uNAG to detect AKI in ICU patients. However, the optimal cut-off value of uNAG in detecting AKI could be affected by stress hyperglycemia in non-diabetic patients.

## Additional files

Additional file 1: **Table S1.** Performance of uNAG in detecting AKI in quintile of admission serum glucose concentrations. (DOC 43 kb)

Additional file 2: **Figure S1.** Concentration of uNAG for non-diabetic patients stratified according to admission serum glucose. (TIF 327 kb)

Additional file 3: **Table S2.** AUCs for AKI stratified according to admission serum glucose in diabetic patients. (DOC 35 kb)

Additional file 4: **Table S3.** AUCs for AKI stratified according to HbA1c levels and history of diabetes. (DOC 51 kb)

Additional file 5: **Table S4.** Performance of NAG in detecting AKI by quartile of HbA1c. (DOC 40 kb)

## Abbreviations

5% CI: 95% confidence interval
AKI: Acute kidney injury
APACHE II: Acute Physiology and Chronic Health Evaluation II score
AUC: Area under the receiver operating characteristic curve
BMI: Body mass index
CKD: Chronic kidney disease, defined as baseline estimated glomerular filtration rate < 60 ml/min/1.73 m2
DM: Diabetes mellitus
eGFR: Estimated glomerular filtration rate
ESRD: End-stage renal disease
HbA1c: Glycosylated hemoglobin
ICU: Intensive care unit
n: Sample size
ROC: Receiver operating characteristic curve
RRT: Renal replacement therapy
sCr: Serum creatinine
uNAG: Urinary N-acetyl-β-D-glucosamindase
UP: Urine production first 24 h after admission
