# Supplementary material for: Impact of blood glucose levels on the accuracy of urinary N-acety-β-D-glucosaminidase for acute kidney injury detection in critically ill adults: a multicenter, prospective, observational study
Source: BMC Nephrol. 2019 May 24;20:186. doi: 10.1186/s12882-019-1381-3 (PMC6534873; doi:10.1186/s12882-019-1381-3)
Supplement: Supplementary file 1 — Table S1. Performance of uNAG in detecting AKI in quintile of admission serum glucose concentrations. (DOC 43 kb) [file 12882_2019_1381_MOESM1_ESM.doc]

Additional table 1. Performance of uNAG in detecting AKI in quintile of admission serum glucose concentrations

| Group | AKI (*n*, %) | AUC-ROC | 95% CI | Cut-off (U/g Cr) | Sensitivity | Specificity |
| --- | --- | --- | --- | --- | --- | --- |
| Total AKI (*n* = 412) | | | | | | |
| Quintile I (*n* = 286) | 48 (16.8) | 0.656±0.045 | 0.598-0.711 | 19.45 | 0.792 | 0.487 |
| Quintile II (*n* = 286) | 53 (18.5) | 0.623±0.039 | 0.564-0.680 | 15.30 | 0.943 | 0.322 |
| Quintile III (*n* = 288) | 77 (26.7) | 0.650±0.036 | 0.591-0.705 | 24.14 | 0.610 | 0.630 |
| Quintile IV (*n* = 289) | 90 (31.1) | 0.640±0.036 | 0.582-0.695 | 33.78 | 0.567 | 0.694 |
| Quintile V (*n* = 288) | 144 (50.0) | 0.648±0.033 | 0.590-0.703 | 31.56 | 0.694 | 0.597 |
| Severe AKI (*n* = 109) | | | | | | |
| Quintile I (*n* = 286) | 10 (3.5) | 0.704±0.102 | 0.648-0.756 | 38.37 | 0.700 | 0.790 |
| Quintile II (*n* = 286) | 12 (4.2) | 0.703±0.070 | 0.647-0.756 | 34.13 | 0.667 | 0.675 |
| Quintile III (*n* = 288) | 17 (5.9) | 0.761±0.065 | 0.708-0.809 | 31.65 | 0.765 | 0.716 |
| Quintile IV (*n* = 289) | 29 (10.0) | 0.693±0.047 | 0.636-0.746 | 33.95 | 0.724 | 0.658 |
| Quintile V (*n* = 288) | 41 (14.2) | 0.697±0.041 | 0.640-0.750 | 35.54 | 0.829 | 0.551 |

AUC, area under the receiver operating characteristic curve; AKI, acute kidney injury; n*,* sample size; 95% CI*,* 95% confidence interval. Total AKI:

Quintile I versus Quintile II Z = 0.556, *P* = 0.578;

Quintile I versus Quintile III Z = 0.104, *P* = 0.917;

Quintile I versus Quintile IV Z = 0.280, *P* = 0.779;

Quintile I versus Quintile V Z = 0.145, *P* = 0.885;

Quintile II versus Quintile III Z = 0.507, *P* = 0.612;

Quintile II versus Quintile IV Z = 0.321, *P* = 0.748;

Quintile II versus Quintile V Z = 0.492, *P* = 0.623;

Quintile III versus Quintile IV Z = 0.197, *P* = 0.844;

Quintile III versus Quintile V Z = 0.041, *P* = 0.967;

Quintile IV versus Quintile V Z = 0.166, *P* = 0.868.

Severe AKI:

Quintile I versus Quintile II Z = 0.008, *P* = 0.994;

Quintile I versus Quintile III Z = 0.472, *P* = 0.637;

Quintile I versus Quintile IV Z = 0.098, *P* = 0.922;

Quintile I versus Quintile V Z = 0.064, *P* = 0.949;

Quintile II versus Quintile III Z = 0.609, *P* = 0.542;

Quintile II versus Quintile IV Z = 0.119, *P* = 0.905;

Quintile II versus Quintile V Z = 0.074, *P* = 0.941;

Quintile III versus Quintile IV Z = 0.850, *P* = 0.395;

Quintile III versus Quintile V Z = 0.834, *P* = 0.404;

Quintile IV versus Quintile V Z = 0.064, *P* = 0.949.
