# Supplementary figures and images for: Impact of blood glucose levels on the accuracy of urinary N-acety-β-D-glucosaminidase for acute kidney injury detection in critically ill adults: a multicenter, prospective, observational study
Source: BMC Nephrol. 2019 May 24;20:186. doi: 10.1186/s12882-019-1381-3 (PMC6534873; doi:10.1186/s12882-019-1381-3)

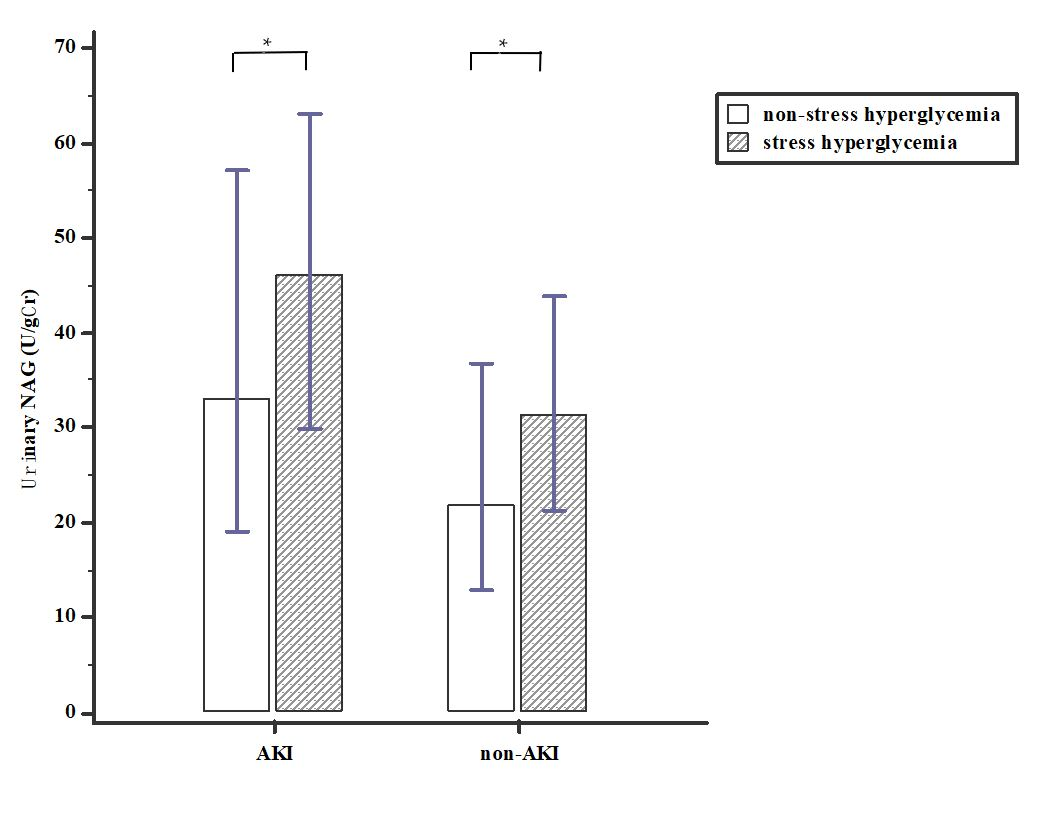

Supplement: Supplementary file 2 — Figure S1. Concentration of uNAG for non-diabetic patients stratified according to admission serum glucose. (TIF 327 kb) [file 12882_2019_1381_MOESM2_ESM.tif]
