# Supplementary material for: Impact of blood glucose levels on the accuracy of urinary N-acety-β-D-glucosaminidase for acute kidney injury detection in critically ill adults: a multicenter, prospective, observational study
Source: BMC Nephrol. 2019 May 24;20:186. doi: 10.1186/s12882-019-1381-3 (PMC6534873; doi:10.1186/s12882-019-1381-3)
Supplement: Supplementary file 3 — Table S2. AUCs for AKI stratified according to admission serum glucose in diabetic patients. (DOC 35 kb) [file 12882_2019_1381_MOESM3_ESM.doc]

Additional table 4**.** AUCs for AKI stratified according to admission serum glucose in diabetic patients

| Glucose (mg/dL) | AKI (*n*, %) | AUC-ROC | 95% CI | Cut-off (U/g Cr) | Sensitivity | Specificity |
| --- | --- | --- | --- | --- | --- | --- |
| Total AKI (*n* = 108) | | | | | | |
| <200 (*n* = 156) | 64 (41.0) | 0.653±0.045 | 0.572-0.727 | 28.56 | 0.672 | 0.609 |
| ≥200 (*n* = 69) | 44 (63.8) | 0.649±0.073 | 0.524-0.760 | 28.03 | 0.750 | 0.600 |
| Severe AKI (*n* = 35) | | | | | | |
| <200 (*n* = 156) | 21 (13.5) | 0.712±0.056 | 0.634-0.781 | 32.01 | 0.857 | 0.600 |
| ≥200 (*n* = 69) | 14 (20.3) | 0.715±0.071 | 0.594-0.817 | 33.95 | 0.857 | 0.618 |

AUC, area under the receiver operating characteristic curve; AKI, acute kidney injury; *n,* sample size; 95% CI*,* 95% confidence interval. Total AKI:

≥200 mg/dL versus <200 mg/dL Z = 0.047, *P* = 0.963.

Severe AKI:

≥200 mg/dL versus <200 mg/dL Z = 0.033, *P* = 0.974.
