# Supplementary material for: Impact of blood glucose levels on the accuracy of urinary N-acety-β-D-glucosaminidase for acute kidney injury detection in critically ill adults: a multicenter, prospective, observational study
Source: BMC Nephrol. 2019 May 24;20:186. doi: 10.1186/s12882-019-1381-3 (PMC6534873; doi:10.1186/s12882-019-1381-3)
Supplement: Supplementary file 4 — Table S3. AUCs for AKI stratified according to HbA1c levels and history of diabetes. (DOC 51 kb) [file 12882_2019_1381_MOESM4_ESM.doc]

Additional table 2. AUCs for AKI stratified according to HbA1c levels and history of diabetes

| Group | AKI (*n*, %) | AUC-ROC | 95% CI | Cut-off (U/g Cr) | Sensitivity | Specificity |
| --- | --- | --- | --- | --- | --- | --- |
| Total AKI (*n* = 412) |  |  |  |  |  |  |
| recognized diabetes (*n* = 98) | 53 (41.7) | 0.675±0.055 | 0.573-0.767 | 28.29 | 0.698 | 0.644 |
| patients without known prior history of diabetes | | | | | | |
| HbA1c ≥6.5% (*n* = 127) | 55 (43.3) | 0.649±0.049 | 0.559-0.731 | 29.89 | 0.709 | 0.597 |
| 5.7% ≤HbA1c <6.5% (*n* = 513) | 142 (27.7) | 0.645±0.026 | 0.602-0.687 | 27.23 | 0.662 | 0.561 |
| HbA1c <5.7% (*n* = 699) | 162 (23.2) | 0.659±0.025 | 0.621-0.693 | 29.18 | 0.568 | 0.676 |
| Severe AKI (*n* = 109) |  |  |  |  |  |  |
| recognized diabetes (*n* = 98) | 17 (17.3) | 0.731±0.068 | 0.631-0.815 | 33.86 | 0.882 | 0.630 |
| patients without known prior history of diabetes | | | | | | |
| HbA1c ≥6.5% (*n* = 127) | 18 (14.2) | 0.704±0.053 | 0.617-0.782 | 32.98 | 0.833 | 0.578 |
| 5.7% ≤HbA1c <6.5% (*n* = 513) | 34 (6.6) | 0.700±0.039 | 0.658-0.739 | 32.98 | 0.735 | 0.628 |
| HbA1c <5.7% (*n* = 699) | 40 (5.7) | 0.734±0.044 | 0.700-0.766 | 38.82 | 0.650 | 0.772 |

AUC, area under the receiver operating characteristic curve; AKI, acute kidney injury; HbA1c, glycosylated hemoglobin; n, sample size; 95% CI*,* 95% confidence interval.

Total AKI:

recognized diabetes versus undetected diabetes Z = 0.354, *P* = 0.723;

recognized diabetes versus prediabetes Z = 0.493, *P* = 0.622;

recognized diabetes versus normal glycemic status Z = 0.266, *P* = 0.790;

undetected diabetes versus prediabetes Z = 0.072, *P* = 0.943;

undetected diabetes versus normal glycemic status Z = 0.183, *P* = 0.855;

prediabetes versus normal glycemic status Z = 0.388, *P* = 0.698.

Severe AKI:

recognized diabetes versus undetected diabetes Z = 0.313, *P* = 0.754;

recognized diabetes versus prediabetes Z = 0.396, *P* = 0.692;

recognized diabetes versus normal glycemic status Z = 0.037, *P* = 0.970;

undetected diabetes versus prediabetes Z = 0.060, *P* = 0.952;

undetected diabetes versus normal glycemic status Z = 0.432, *P* = 0.665;

prediabetes versus normal glycemic status Z = 0.575, *P* = 0.565.
