# Supplementary material for: Impact of blood glucose levels on the accuracy of urinary N-acety-β-D-glucosaminidase for acute kidney injury detection in critically ill adults: a multicenter, prospective, observational study
Source: BMC Nephrol. 2019 May 24;20:186. doi: 10.1186/s12882-019-1381-3 (PMC6534873; doi:10.1186/s12882-019-1381-3)
Supplement: Supplementary file 5 — Table S4. Performance of NAG in detecting AKI by quartile of HbA1c. (DOC 40 kb) [file 12882_2019_1381_MOESM5_ESM.doc]

Additional table 3**.** Performance of NAG in detecting AKI by quartile of HbA1c

| Group | AKI (*n*, %) | AUC-ROC | 95% CI | Cut-off (U/g Cr) | Sensitivity | Specificity |
| --- | --- | --- | --- | --- | --- | --- |
| Total AKI (*n* = 412) |  |  |  |  |  |  |
| Quartile I (*n* = 280) | 60 (21.4) | 0.642±0.043 | 0.583-0.698 | 34.84 | 0.500 | 0.786 |
| Quartile II (*n* = 424) | 106 (25.0) | 0.660±0.030 | 0.613-0.705 | 23.08 | 0.698 | 0.528 |
| Quartile III (*n* = 356) | 90 (25.3) | 0.658±0.032 | 0.606-0.707 | 20.60 | 0.822 | 0.462 |
| Quartile IV (*n* = 377) | 156 (41.4) | 0.648±0.029 | 0.597-0.696 | 32.71 | 0.615 | 0.638 |
| Severe AKI (*n* = 109) |  |  |  |  |  |  |
| Quartile I (*n* = 280) | 15 (5.4) | 0.756±0.074 | 0.702-0.805 | 48.28 | 0.600 | 0.879 |
| Quartile II (*n* = 424) | 27 (6.4) | 0.735±0.055 | 0.691-0.777 | 40.77 | 0.667 | 0.771 |
| Quartile III (*n* = 356) | 19 (5.3) | 0.776±0.049 | 0.729-0.819 | 32.98 | 0.842 | 0.653 |
| Quartile IV (*n* = 377) | 48 (12.7) | 0.672±0.036 | 0.622-0.719 | 32.98 | 0.771 | 0.581 |

AUC, area under the receiver operating characteristic curve; AKI, acute kidney injury; HbA1c, glycosylated hemoglobin; *n,* sample size; 95% CI*,* 95% confidence interval.

Total AKI:

Quartile I versus Quartile II Z = 0.342, *P* = 0.733;

Quartile I versus Quartile III Z = 0.297, *P* = 0.766;

Quartile I versus Quartile IV Z = 0.116, *P* = 0.908;

Quartile II versus Quartile III Z = 0.046, *P* = 0.964;

Quartile II versus Quartile IV Z = 0.290, *P* = 0.772;

Quartile III versus Quartile IV Z = 0.234, *P* = 0.815.

Severe AKI:

Quartile I versus Quartile II Z = 0.229, *P* = 0.819;

Quartile I versus Quartile III Z = 0.226, *P* = 0.821;

Quartile I versus Quartile IV Z = 1.023, *P* = 0.306;

Quartile II versus Quartile III Z = 0.560, *P* = 0.576;

Quartile II versus Quartile IV Z = 0.961, *P* = 0.336;

Quartile III versus Quartile IV Z = 1.706, *P* = 0.088.
